# Windswept deformities of the knee are challenging to manage

**DOI:** 10.1186/s43019-020-00062-3

**Published:** 2020-08-31

**Authors:** Suresh Babu, Abhishek Vaish, Raju Vaishya

**Affiliations:** Department of Orthopaedics and Joint Replacement Surgery, Indraprastha Apollo Hospitals, Sarita Vihar, New Delhi, 110076 India

**Keywords:** Windswept deformities, Varus deformity, Valgus deformity, Knee, Arthroplasty, Simultaneous bilateral

## Abstract

**Background:**

Little has been published about TKA in windswept deformities of the knees where combined varus and valgus deformities present in the same patient. Windswept deformities present with unique problems and must be addressed as two halves of a complex entity. Through this review we aim to understand the interrelation between the deformities, examine outcomes following simultaneous bilateral total knee arthroplasty in windswept deformities, and develop an algorithm for the management of windswept deformities by total knee arthroplasty.

**Methods:**

An extensive online literature search for the keywords yielded 31 articles on which we based our review. Articles were analyzed in context to our research questions and are presented in a tabular format for quick reference and a better perspective.

**Results:**

The abnormal biomechanics and force moment of the knee cause progressive arthritis of the knee. The valgus deformity usually precedes a varus deformity on the contralateral knee in windswept deformities. Correct restoration of mechanical tibiofemoral angles by individualizing valgus correction angles have better outcomes after TKA.

**Conclusion:**

A well-planned and judiciously executed simultaneous bilateral total knee replacement can offer distinct advantages to the patient and surgeon and provides optimum utilization of time and resources in the management of windswept knees.

## Introduction

Windswept deformities (WSD) of the knee are not common presentations and pose unique challenges during total knee arthroplasty (TKA). In addition to resurfacing the arthritic surfaces of the joint, restoration of the normal biomechanics of the knee is essential [1]. WSD present a scenario with the knees at two extremes of the deformity spectrum in the coronal plane, and each extreme shows varied bony and soft tissue insufficiencies [2]. The etiopathology of the deformities is different and also needs to be addressed [3]. In a WSD, there is primarily medial compartment osteoarthritis (OA) on the side of varus deformity [2, 3] and lateral compartment OA on the side of the valgus deformity. The soft tissues on the medial side of the knee are contracted and need to be released in a varus knee [3], whereas in a valgus knee, the soft tissues on the lateral side of the knee are contracted and require release [1–3]. There are varying degrees of patellofemoral arthritis, and patellar tracking should be optimized to obtain superior outcomes, which in many instances, especially with valgus knees, may require a lateral retinacular release [4–6]. There is a paucity of literature to help understand the influence of individual deformities on the contralateral knee and their importance in management by total knee replacement. Whether the deformities represent a continuum in the spectrum of windswept knees and what the management protocols should be are also unclear.

We therefore reviewed the literature to identify the challenges to TKA in WSD and to contribute to an understanding of the following:

1. Whether an interrelation exists between the deformities and whether the effect of kinematics is mutually inclusive
2. The outcomes with simultaneous bilateral total knee arthroplasty (SBTKA)
3. The need for constrained designs, stem extensions, and additional procedures
4. The possibility of formulating an algorithm for total knee arthroplasty in windswept deformities

## Materials and methods

**Fig. 1:**
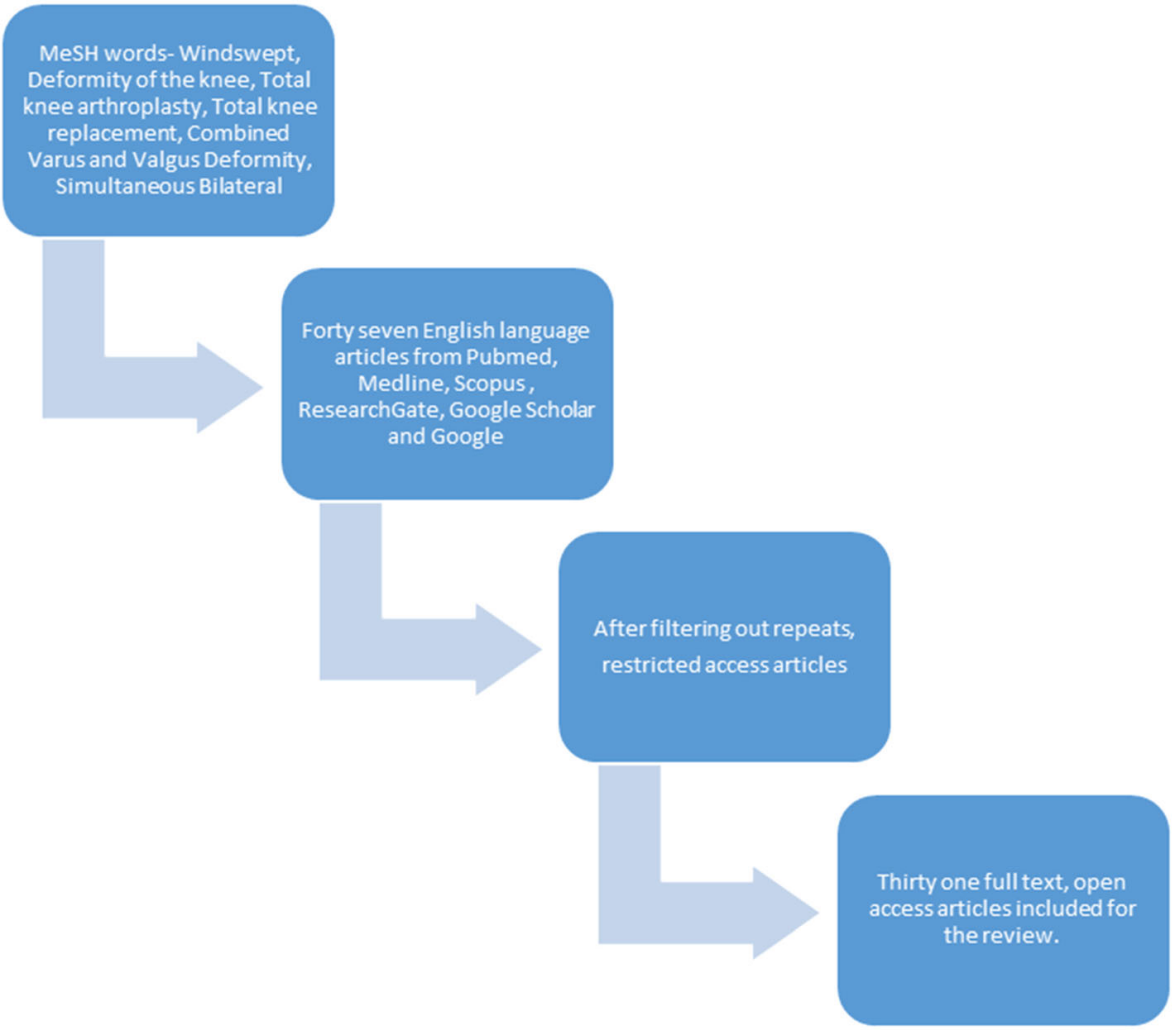
Flow chart of literature search

We did a comprehensive literature search of the indexed databases including PubMed, ResearchGate, Google Scholar, Scopus, Medline, and Google Search using the MeSH words, “Windswept, Deformity of the knee, Total Knee Arthroplasty, Total Knee Replacement, Combined Varus-Valgus, Simultaneous bilateral total knee arthroplasty.” We could find a total of 47 articles in the literature. After filtering for open access, complete text, and English language articles, we were able to reference 31 articles for our study (Fig. 1). Citations were obtained for the selected articles in the required format to create the reference section.

All the studies selected were analyzed in context to the aims of this study. We tabulated highly relevant studies and collated details of the author, journal, year of publication, level of evidence, and the conclusions drawn from the studies (Table 1). The articles were segregated and analyzed relative to biomechanics and gait, clinical significance of WSD, implications of WSD in planning for TKA, intra-operative planning of valgus correction angle (VCA), and clinical outcomes following bilateral staged or SBTKA. Adequate and appropriate referencing was done in the preparation of the manuscript. An algorithm was also prepared.

**Table 1:**
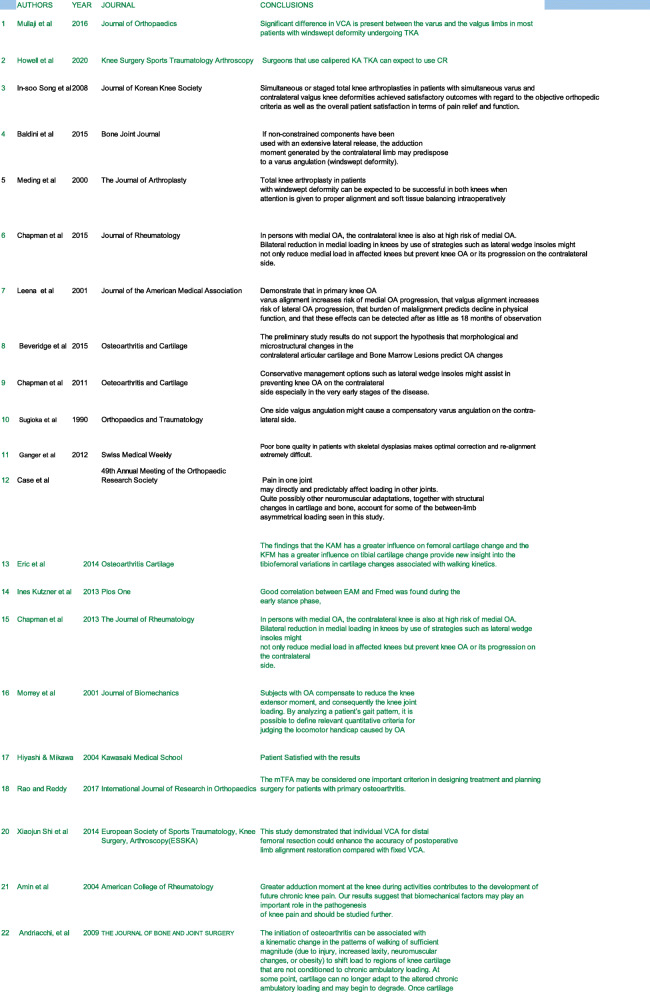
Studies and their findings related to windswept knees

## Results

### Biomechanics and gait

Kutzner et al. [7] noticed a correlation between knee adduction moment and medial contact force during gait using telemetric data transmission in the instrumented knee (following TKA) and found a good correlation between external adductor moment and medial joint force. Amin et al. [8], in their study of knee adduction moment (KAM) and development of chronic knee pain in 132 elders found that a greater adduction moment at the knee during activities contributes to the development of future chronic knee pain and suggested that biomechanical factors may play an important role in the pathogenesis and progression of knee pain. This finding has implications in our understanding of the development of knee osteoarthritis, which begins with abnormal alignment (deformity) and the increased force moments around the knee that lead to degenerative changes. Andriacchi et al. and Beveridge et al. [9–11] have studied the effects of gait mechanics on healthy cartilage morphology and knee OA and concluded that adduction moment during walking can be predictive of the clinical outcomes of treatment. Morrey et al. [12], in their study of knee kinematics in 139 patients with grade two knee OA, reported that subjects with OA compensate to reduce the knee extensor moment and, consequently, affect their joint loading and gait patterns. These parameters are quantitatively evaluated in measuring the locomotor handicap caused by OA. Based on these studies, this malalignment, by inference, is an essential prerequisite for the development of osteoarthritis and deformities in windswept deformities, which mutually perpetuate and aggravate osteoarthritis of the contralateral knee.

### Clinical significance of windswept deformities

Sugioka et al. [13], in a retrospective study, reviewed five adult patients who had a WSD. They made an important observation that one side valgus angulation might cause a compensatory varus angulation on the contralateral side, although this finding was not substantiated by any further studies. Case et al. [14], in their presentation on the gait observational data in 53 patients, have opined that pain in one joint may directly and predictably affect loading in other joints. Rao and Reddy [15], in their study on the association of frontal plane tibiofemoral alignment with knee pain in 314 knees (195 patients), found a positive correlation between coronal tibiofemoral angles and pain in primary OA. Leena Sharma [16] observed that the burden of malalignment of the knees predicts a decline in physical function and that these effects can be detected as early as 18 months.

### Implications for intraoperative planning of valgus correction angle

Mullaji et al. [17] studied the variation in femoral valgus correction angle (VCA) between the two limbs in patients with windswept deformity undergoing TKA and found that VCA in varus knees was significantly higher compared to mean VCA in the valgus knees. Shi et al. [18], in their large study on the accuracy of using individualized valgus correction angle during TKA for varus and valgus deformities of the knee, concluded that individual correction angles improve the accuracy of postoperative limb alignment after TKA compared with using fixed valgus correction guides. Nam et al. [19], in a retrospective review of 320 consecutive patients, found that the use of a variable distal femur resection angle improves femoral component alignment after TKA. Similarly, Zhou K et al. [20], in a radiological study, showed that individual VCA for distal femoral resection could achieve better postoperative alignment accuracy and fewer outliers of limb and femoral component malalignment in the coronal plane.

### Clinical outcomes following bilateral TKA in windswept deformities

Howell et al. [21], in a prospective review of 19 patients who underwent bilateral TKA in WSD, looked into the level of implant constraint, outcome scores, and alignment after bilateral, callipered, kinematically aligned TKA and observed that no knees required semi-constrained implant or posterior cruciate ligament release, and a short tibial stem extension was used in only one valgus knee. They did not find any difference in the median postoperative “Forgotten Joint Scores” and “Oxford Knee Scores” between paired varus and valgus knees. Their management was a staged procedure of doing the knees at separated intervals and sequences, depending on the severity. They achieved one degree or less in the mean difference in postoperative distal lateral femoral angle and the proximal medial tibial angle between the varus and valgus knees using cruciate-retaining implants. Song et al. [22], in their study of 14 patients of WSD who underwent SBTKA, found comparable knee scores for both deformities at an average follow-up of 18.4 months. However, Baldini et al. [23] cautioned against not using constrained components in valgus knees that needed extensive lateral release, suggesting that the adduction moment generated by the contralateral knee can predispose to varus angulation in the operated knee. Meding et al. [24] studied 20 knees with WSD undergoing bilateral TKA (simultaneous in 18 and staggered in two patients) and found the procedure to be successful in both knee groups (varus/valgus) and that proper attention to alignment and soft tissue balancing intraoperatively are essential. Hiyashi and Mikawa [25], in their case report of a 63-year-old female with WSD treated by bilateral TKA, reported a high satisfaction level in the patient following the procedure. Ganger et al. [22], in a case report of WSD in a patient with skeletal dysplasia, commented on the poor bone quality in these patients, which made optimal correction and realignment difficult.

## Discussion

**Fig. 2:**
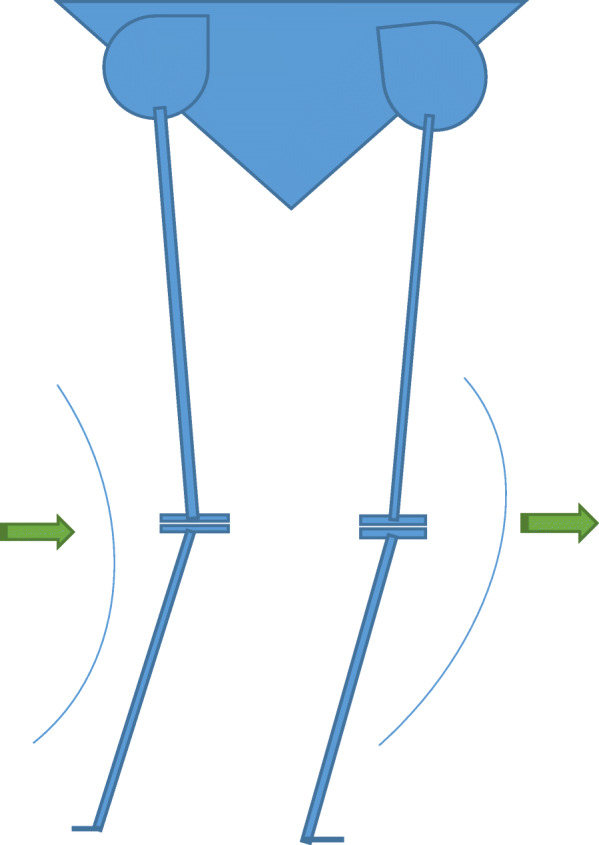
Diagrammatic representation of a Windswept deformity with varus and valgus

**Fig. 3:**
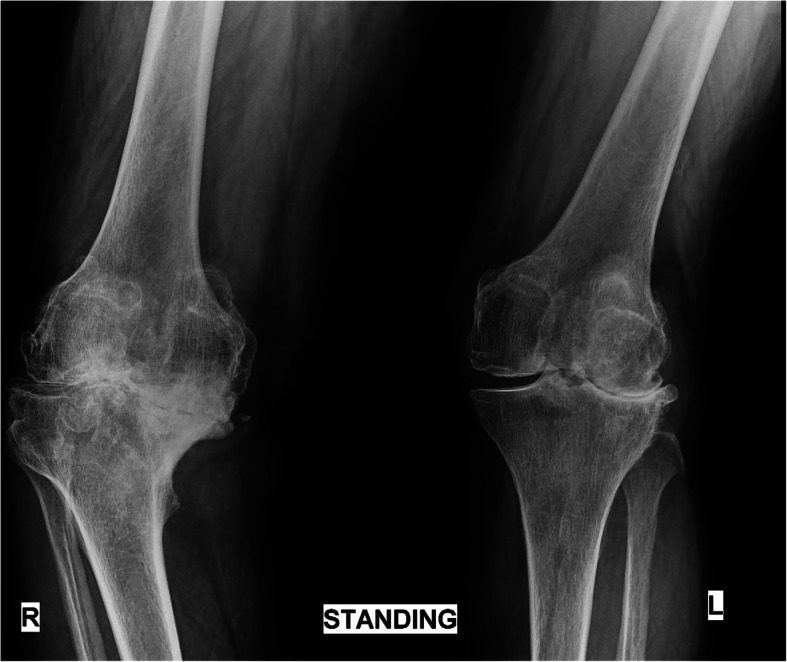
X-ray of a windswept deformity with combined varus and valgus knees

**Fig. 4:**
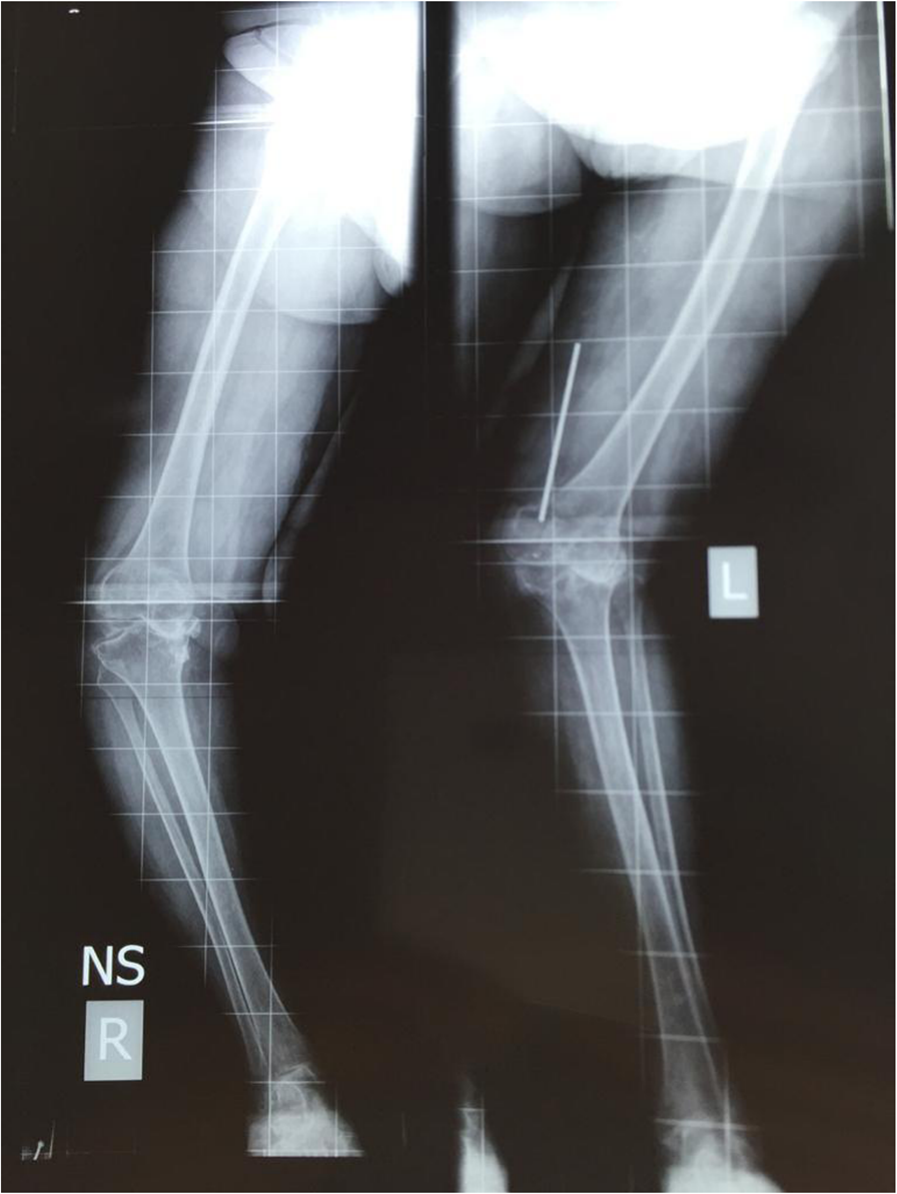
Scanogram of windswept knees

Windswept deformities (WSD) of the knee are so named as to describe varus deformity on one side with valgus on the contralateral side [17]; for example, if the wind blew across the knees from left to right, a valgus deformity would be produced on the left and a varus on the right (Fig. 2, Fig. 3). In a WSD, there is a predominantly medial compartment OA on the side of varus deformity and lateral compartment OA on the side of the valgus deformity [17, 24] (Fig. 4).

Although no etiology exists as a specific cause of windswept deformities, these deformities can be seen in inflammatory arthritides like rheumatoid arthritis, skeletal dysplasias, sequelae of childhood rickets, lateral femoral condyle dysplasia in one knee, and as a result of the overcorrection of genu varum on one side [24–26]. Malalignment at the knee increases the adduction or abduction moments around the knee and leads to deterioration of the OA changes in the knees. The burden of malalignment is proportionate to the decline in the physical function of the knee [7]. Deformities may be biomechanically interrelated going by the sequence of deformities and loading patterns, and the contralateral knee also can be safely assumed to become symptomatic in time [8–11]. Neglected deformities of the knee with WSD cause significant locomotor disability [12] and correspondingly leads to a prolonged recovery time and therefore should be addressed promptly by structured physical exercise and a muscle strengthening program postoperatively. Valgus in one knee usually proceeds a varus deformity on the contralateral knee and can be mutually compensating [13]. Abnormal loading forces in one knee due to deformity predictably affect loading patterns and loading of the contralateral knee [14]. Conditions with marked deviation in tibiofemoral alignment angles, as seen in WSD, produce considerable morbidity and abnormality in gait patterns [15]. Abnormal force moments around the knee influence progression of pain and OA of the contralateral knee [16]. Although the procedure of TKA is successful with both primary posterior stabilized and cruciate-retaining implants, the surgeon should not hesitate to increase the level of constraint and to use stem extensions if the situation so warrants [21]. Outcomes with single or staged TKA are good and comparable with TKA for bilateral knee OA without WSD [21–25]. Skeletal dysplasia, condylar hypoplasia, rheumatoid arthritis, and bony defects present both a defective and deficient bone stock during TKA, which makes the surgery challenging [26].

### Management

Windswept deformities present a difficult surgical preposition in terms of [2, 3, 17, 24] of the following:

- bilateral affection
- grotesque deformities
- bone defects
- ligamentous laxity
- locomotor disability
- poor bone stock and quality as a result of dysplasia and inflammatory arthritis or sequelae of childhood rickets
- Limb length discrepancies
- peroneal nerve injury, in the valgus knee

Hence, the WSD should be approached as a single entity, and each knee should be considered as one-half of the problem. Treating the problem in its entirety makes sound clinical sense due to the following perspectives.

#### Anatomy and biomechanical perspective

The WSD presenting with significant malalignment and altered biomechanics with a deformity in one knee precipitating and aggravating the disease and symptoms of the contralateral knee [7] is challenging. Abnormal force moments (adductor moment in varus and abductor moment in valgus) have a cascading effect on the gait pattern, joint loading, muscle conditioning, and energy consumption [8]. The main component of the deformities is bony, with ligamentous attenuation being contributory. Therefore, the deformities in WSD are mutually inclusive and need to be addressed as a single problem requiring bilateral TKA [9, 10].

#### Surgical perspective

We draw from our review of literature and experience that SBTKA in the medically fit group of patients is advantageous for the following reasons:

- It prevents duplication of perioperative procedures and scheduling of operation theatre rosters in a busy set-up [27].
- It avoids the cumulative risk of repeated anesthesia and physiological stress of surgery [28].
- It provides an opportunity to compare the alignment and of both limbs and equalize limb length during surgery while both are sterile-draped [29].
- It provides an opportunity for using autologous bone available from bony resections to build the bony defects [2].

#### Patient perspective

Correcting the WSD at a single procedure is a patient-friendly option for the following reasons:

- It is less stressful for the patient [28].
- It avoids the antecedent complications of repeated surgery [28].
- It provides replacement of both knees together (albeit a few months if planned as a staggered procedure) and can be done under a single admission and anesthesia, thereby saving on the financial overrun [30].
- An integrated rehabilitation program for both the knees can be followed [30].
- It optimizes the patient-support services in the family and community.

#### Algorithm

**Fig. 5:**
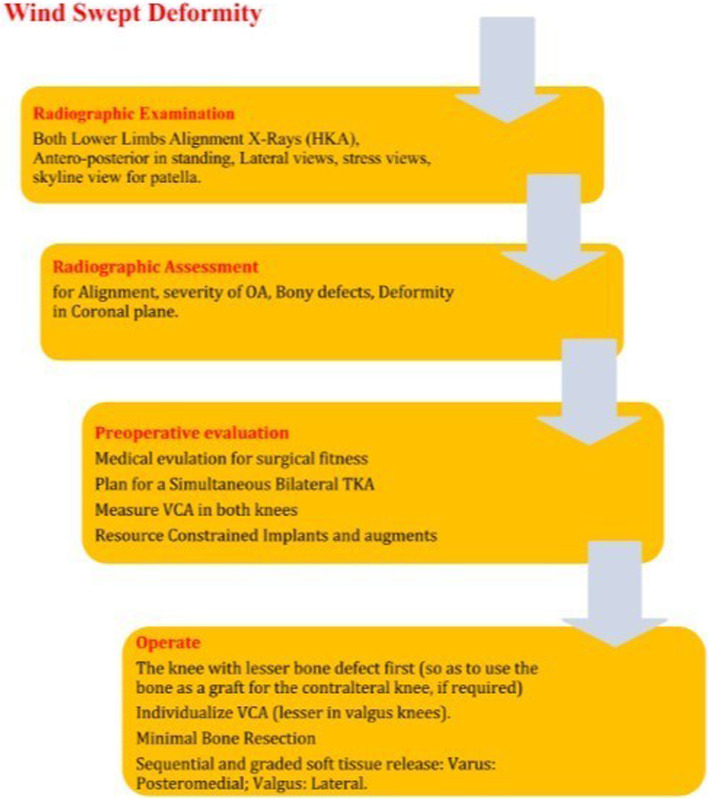
Algorithm for the management of windswept knees

Based on our experience [31] and the review of the literature, we propose an algorithm for TKA in the management of WSD of the knees (Fig. 5).

### Limitations

This review lacks the robustness due to an absolute paucity of literature that has addressed WSD in totality and as different facets of a single complex deformity. Contemporary literature on TKA in WSD is scarce, and therefore, the results from the limited studies available cannot be adequately validated.

## Conclusion

Knee osteoarthritis with windswept deformities are mutually inclusive and have a compounding effect. The valgus correction angle should be customized to the deformity, and prompt correction of the tibiofemoral angle should be achieved. The deformities in windswept knees are troublesome for the patients and challenging for the surgeons to manage. Outcomes following SBTKA have shown excellent and comparable results to staged procedures and the arthritic knees without WSD. An algorithm for the management of WSD has been presented herewith. A well-planned and judiciously executed SBTKA in the medically fit group of patients offers distinct advantages to the patient and surgeon and provides for optimum utilization of time and resources.

## Data Availability

As this is a review article, any data quoted in the manuscript was obtained from online databases and is adequately referenced. Table 1 and Fig. 1 are original reproductions; patient photograph and X-rays are sourced from personal archives.

## Abbreviations

CR: Cruciate retaining
HKA: Hip knee ankle
OA: Osteoarthritis
PS: Posterior stabilized
SBTKA: Simultaneous bilateral total knee arthroplasty
TKA: Total knee arthroplasty
VCA: Valgus correction angle
WSD: Windswept deformity
